# Identification of the Bisphenol A (BPA) and the Two Analogues BPS and BPF in Cryptorchidism

**DOI:** 10.3389/fendo.2021.694669

**Published:** 2021-07-14

**Authors:** Marta Diana Komarowska, Kamil Grubczak, Jan Czerniecki, Adam Hermanowicz, Justyna Magdalena Hermanowicz, Wojciech Debek, Ewa Matuszczak

**Affiliations:** ^1^ Department of Pediatric Surgery and Urology, Faculty of Medicine, Medical University of Bialystok, Białystok, Poland; ^2^ Department of Regenerative Medicine and Immune Regulation, Medical University of Bialystok, Białystok, Poland; ^3^ Department of Biology and Pathology of Human Reproduction, Institute of Animal Reproduction and Food Research Polish Academy of Sciences, Olsztyn, Poland; ^4^ Department of Pharmacodynamics, Medical University of Bialystok, Białystok, Poland; ^5^ Department of Clinical Pharmacy, Medical University of Bialystok, Białystok, Poland

**Keywords:** bisphenol (BPA), bisphenol S (BPS), bisphenol F (BPF), cryptorchidism, children

## Abstract

**Objective:**

to explore the association of plasma concentrations of bisphenol A (BPA), bisphenol S (BPS), and bisphenol F (BPF) with unilateral cryptorchidism. In addition, to analyze selected demographic and intraoperative characteristics.

**Design:**

Retrospective analysis to determine plasma concentrations of total BPA, BPS and BPF using gas chromatography - mass spectrometry (GC-MS) among prepubertal boys with cryptorchidism and prebupertal male control subjects. During operation, the size, turgor and location of the cryptorchid testes were assessed.

**Main Outcome Measure:**

Plasma concentrations of total BPA, BPS and BPF.

**Results:**

In children with cryptorchidism, plasma levels of BPA, BPS and BPF were significantly higher compared to the control subjects. For BPA, it was: median value: 9.95 ng/mL *vs*. 5.54 ng/mL, p<0.05. For BPS, it was: median value: 3.93 ng/mL *vs*. 1.45 ng/mL, p<0.001. For BPF, it was: median value: 3.56 ng/mL *vs*. 1.83 ng/mL, p<0.05. In cryptorchid group, BPA was detected in 61.4% samples, BPS in 19.3% and BPF in 19.3%. All the three bisphenols were detected in plasma samples of both the healthy subjects and the study cohort. In the latter group, we found significant higher levels of BPA in boys from urban areas. We found a weak positive correlation between the levels of BPS and BPF and reduced turgor of the testes. Furthermore, results showed weak positive correlations between BPA and BPS levels and the age of the children as well as between BPS and BPF concentrations and the place of residence.

**Conclusions:**

Results provide a first characterization of prepubertal boys suffering from cryptorchidism and exposed to different kind of bisphenols. Our study suggests that cryptorchid boys are widely exposed to BPA and, to a lesser extent, also to its alternatives, such as BPS and BPF.

## Introduction

At the moment humans are exposed to numerous chemicals. Endocrine disrupting chemicals (EDCs) are exogenous substances that could interfere with hormonal system (1). Bisphenol A (BPA), a xenoestrogen, is one of the most abundant EDCs. Predicted worldwide consumption of BPA in 2022 will be approximately 10.6 million metric tons (2). Results of the surveys regarding the harmful effect of BPA on humans tend to be contradictory, but it is worth to note that the European Union, the United States, and Canada have been gradually banning BPA from baby products, such as bottles and sippy cups. In response to many reports on the harmful effects of BPA on human health, producers are trying to replace one type of bisphenol with another.

Bisphenol S (BPS) (4,4′-sulfonylbisphenol) and bisphenol F (BPF) (4,4′-dihydroxydiphenyl-methane) are analogues of the ubiquitous xenoestrogen 2,2-bis (4-hydroxyphenol) propane) (3). Their chemical structures with simple side chain modifications are similar to those of the most common and notorious BPA. Assessment of the impact of bisphenol A on humans is difficult. However, it seems that especially children are sensitive to BPA, primarily because of long-term, sometimes even prenatal, exposure (4). We have less knowledge about the toxic effect of both analogues, but according to current *in vitro* and *in vivo* results, both of the new phenols could have similar hazardous implications for human health.

The etiology of cryptorchidism, the most common male genital defect is unknown; hence it cannot be prevented. In most cases, cryptorchidism is idiopathic (5). Probably, the etiology is multifactorial, and hormonal, genetic and environmental factors may regulate testicular development and natural descent (5). This is especially important in the face of the present crisis of the reproductive health of men (6). The putative, individual factors that have some association with cryptorchidism are being small for gestational age, low birth weight, prematurity (7), or maternal smoking (8). Researchers have suggested that environmental factors, such as EDCs, could disturb the natural process of testicular descent. BPA has the potential to bind estrogen receptors ERα and ERβ (9). These both receptors were found in the mesothelial layer, stromal cells, and the endothelial layer of paratesticular tissues of normal and undescended testes (10). Delayed treatment of cryptorchidism is a cause of infertility. Recent evidence suggests that BPA could also increase the risk of male infertility (11, 12).

The assumption behind the introduction of BPA substitutes was their neutral nature and significantly lower toxicity. The rapid and widespread introduction of new bisphenol analogues to production impairs further analyses. The new bisphenols, e.g. BPS and BPF, may mutually interact with one another, but also with other EDCs. It is difficult to predict how these structurally similar chemicals can influence each other and bind with the receptors as a mixture. Individually, EDCs may not cause side effects, but it appears that both animals and humans are exposed to low-dose mixtures of different chemicals, which can lead to “cocktail effects” (13). It needs to be underlined that the consequences of human exposure to EDCs are difficult to determine, because of time-related changes. Sources of bisphenols are diverse and urinary excretion of these EDCs is quite rapid (14). Nevertheless, some authors suggest that the side effects of BPA may be transmitted to subsequent generations (15). There are alarming reports that BPA could induce epigenetic modifications and affect transgenerational inheritance (16, 17).

In this study, we investigated the levels of BPS, BPF and BPA in plasma of children with unilateral congenital cryptorchidism. In addition, we analyzed the demographics of the patients and the intraoperative characteristics of undescended testes. According to our knowledge, our study is the first survey on plasma levels of these three phenols in a pediatric population with cryptorchidism.

## Material and Methods

### Patients

We analyzed 98 children with congenital unilateral cryptorchidism, aged 1–4 years, and 19 healthy boys, without any disorders of the testes, at a comparable age of 1–4 years, who had been admitted for herniotomy. It was a prospective study, conducted on patients of the Pediatric Surgery and Urology Department, Medical University of Bialystok, between 2017 and 2018. In both groups, children had no additional chronic conditions and were not undergoing hormonal treatment. Additionally, we analyzed potentially risk of cryptorchidism: gestational age at birth, birth weight, maternal smoking, maternal alcohol consumption and maternal hormonal treatment during pregnancy. The plasma samples were taken on the day of hospital admission, before the operation. The size, position (inguinal canal, abdominal cavity) and turgor (normal, reduced) of the undescended testicles were examined during orchiopexy. Patients were divided into two groups: 0-24 months of age and 24-48 months of age, and their area of residence recorded as urban or rural.

### Methods

4 mL venous blood samples were taken in the morning on the day of surgery and collected in EDTA glass tubes. Then, the blood samples were centrifuged, and the plasma was frozen to −80°C and stored for further analyses.

### Chemicals

BPA (99.9%), pyridine (99.8%), BPAd16 (98%), N,O-bis(trimethylsilyl)trifluoroacetamide (BSTFA), ammonium acetate (BioXtra ≥ 98%), acetonitrile (anhydrous, 99.8%), and water (Chromasolv, HPLC grade) were purchased from Sigma-Aldrich (Steinheim, Germany). Chloroform (99%, GC grade) was obtained from J. T. Baker (Gliwice, Poland). Individual stock solutions of BPA, BPS, BPF and BPAd16 at the concentration of 10 mg/L were dissolved in acetonitrile and stored at −20°C.

### Determination of Total BPS, BPF and BPA

The collected samples were kept at -80°C, thawed at room temperature, and briefly vortexed before analysis. 300 µL aliquots were transferred to polypropylene tubes and fortified with 20 ng of BPA d16 and 30 µL of β-glucuronidase (Sigma Aldrich, Stenheim, Germany) (2,000 IU dissolved in 1M ammonium acetate buffer pH=6.1). The samples were incubated overnight at 37°C. Then, 150 µl of chloroform and 50 µl of acetonitrile were added to the 200 µl aliquots. The tubes were briefly vortexed, sonicated for 1 min in an ice bath and centrifuged for 1 min at 5,000 rpm and 4°C. The 100 µl of lower organic phase separated by centrifugation were transferred into glass vials and evaporated to dryness, using an Eppendorff vacuum concentrator. Then 50 µl of pyridine and 50 µl of BSTFA were added to each sample for derivatisation prior to gas chromatography. GC-MS (gas chromatography mass spectrometry) analysis was performed using Pegasus 4D GCxGC-TOFMS (LECO Corporation St. Joseph, USA). For the analysis, a 30 m x 0.25 mm, 25 µm film thickness capillary column (SGE Analytical Science Ringwood, Australia) was used, under a flow of ultrapure helium 1.0 ml/min and an inline oxygen and moisture trap. The column’s temperature was raised from an initial 130°C to 300°C, at rate 10°C/min. 1 µL volume of derivatized sample was injected in splitless mode. The temperature of the injector and MS source was 250°C. After 480 s of solvent delay, the spectra were collected at an acquisition rate of 10 spectra/s. ChromaTOF v. 4.51.6.0 software was used for instrument control, data acquisition and evaluation. The BPA, BPS and BPF concentrations were calculated using calibration curves for concentrations from 0 to 1,000 ng/ml. The limits of quantification (LOQs) were 0.05ng/ml for total BPS, BPF and BPA.

### Study Approvals

This case-control study was approved by the Ethics Committee of the Medical University of Bialystok (No R-I-002/288/2017). All caregivers agreed to participate in the study and signed informed consent.

### Statistical Analysis

The collected data were statistically analyzed with the use of GraphPad Prism 9.0 software (GraphPad Software Inc., San Diego, USA). Both studied groups, the healthy control (HC) subjects and the unilateral cryptorchidism (UC) patients, did not demonstrate normal (Gaussian) distribution – verified by Shapiro-Wilk and D’Agostino & Pearson test. Thus, non-parametric Mann-Whitney test was implemented to evaluate differences between the UC patients and HC subjects, and for the assessment of bisphenol (BP) types within each studied group. Differential stratification of the samples was applied to verify differences in BPs levels in context of age (0-24 or 24-48 months), place of living (urban or rural area), testes turgor (normal or reduced), testes localization (abdominal or inguinal). Furthermore, non-parametric Spearman test was used to establish mutual correlations between tested BPs in control and patient groups separately. Differences between groups were considered statistically significant at a p value of < 0.05, and indicated with asterisks on the graphs: * - p < 0.05, ** - p < 0.01, *** - p <0.001, **** - p < 0.0001. Depending on the correlation coefficient value, associations between selected variables were described as: no/very weak – r = 0-0.3, weak – r = 0.3-0.5, moderate – r = 0.5-0.7, or strong – r = 0.7-1.0 correlation.

## Results

### Bisphenols Plasma Levels

**Figure 1:**
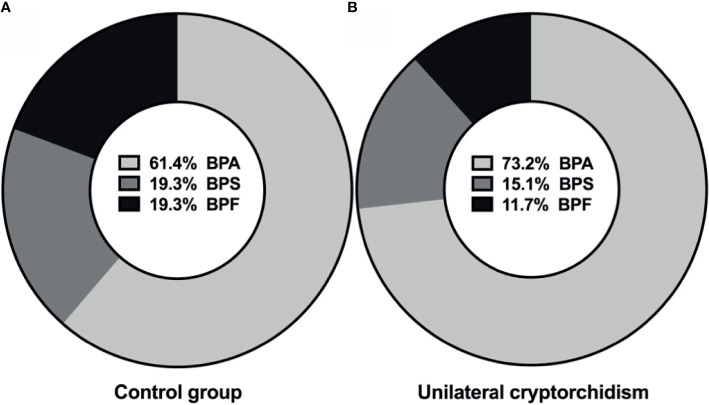
Percentage distributions of bisphenols in plasma of control group **(A)**, and unilateral cryptorchidism **(B)**.

The purpose was to measure the percentage of selected bisphenols (BPs) in the prepubertal boys. All the three bisphenols were detected in plasma in the study and control populations. The most frequently detected compound in the study group was BPA (73.2%), followed by BPS (15.1%) and BPF (11.7%). BPA, BPS and BPF were detected in, respectively, 61.4%, 19.3% and 19.3% of the control group (**Figure 1**). The results suggest that the most common bisphenol in children is BPA. Finding BPS and BPF in the blood is also alarming.

**Figure 2:**
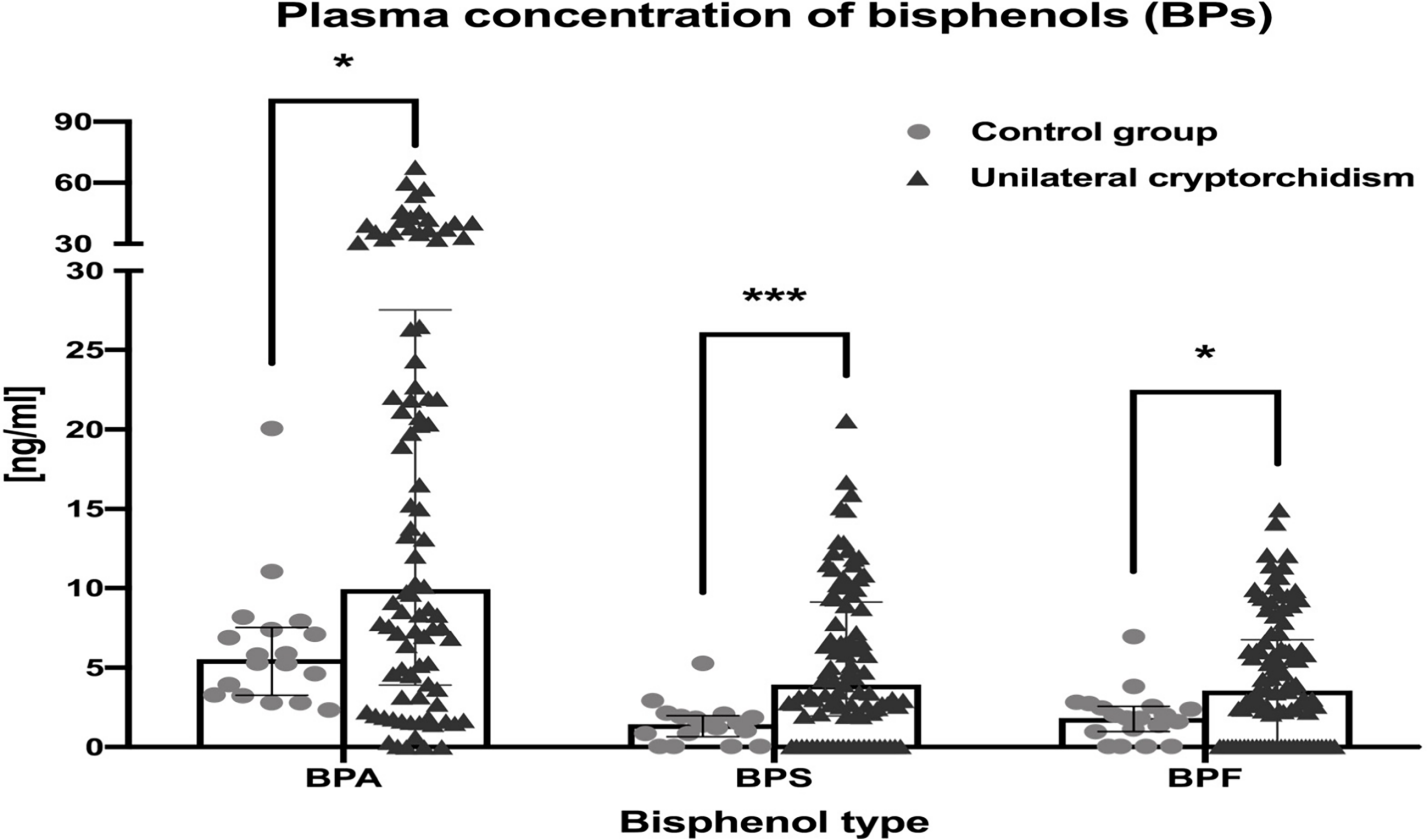
Plasma concentrations of bisphenols (BPs) (*P ≤ 0.05 and ***P ≤ 0.001).

The next goal was to compare the concentration of BPs in healthy boys and children suffered from undescended testis. The highest maximum concentrations of BPA, BPS and BPF in boys with cryptorchidism were 27.54 ng/mL, 9.13ng/ml and 6.76 ng/ml, respectively. In boys with unilateral congenital cryptorchidism, plasma concentrations of all three tested BPs were significantly higher (p value of BPA and BPF < 0.05; p value of BPS < 0.0001) (**Figure 2**). The results indicate that the levels of all three BPs were higher in affected patients.

The following aim was to show numerical values of BPs. For BPA, it was: median value: 9.95 ng/mL; 25^th^-75^th^ percentile [3.91; 27.54] *vs*. 5.54 ng/mL [3.27; 7.53], p<0.05. For BPS, it was: median value: 3.93 ng/mL; 25^th^-75^th^ percentile [1.96; 9.13] *vs*. 1.45 ng/mL [0.65; 1.98], p<0.001. For BPF, it was: median value: 3.56 ng/mL; 25^th^-75^th^ percentile [0.04; 6.76] *vs*. 1.83 ng/mL [0.97; 2.57], p<0.05 (**Table 1**). The results show that the levels of all three BPs were higher in patients with cryptorchidism.

**Table 1:** Bisphenols plasma levels in the healthy control and unilateral cryptorchidism group^a^.

Bisphenols (ng/ml)	Healthy control	Unilateral cryptorchidism
**BPA**	5.54 (3.27; 7.53)	9.95 (3.91; 27.54)
**BPS**	1.45 (0.65; 1.98)	3.93 (1.96; 9.13)
**BPF**	1.83 (0.97; 2.57)	3.56 (0.04; 6.76)

a Data presented as median values with 25^th^ and 57^th^ percentile in the brackets.

### Bisphenols Levels in Reference to Area of Living

**Figure 3:**
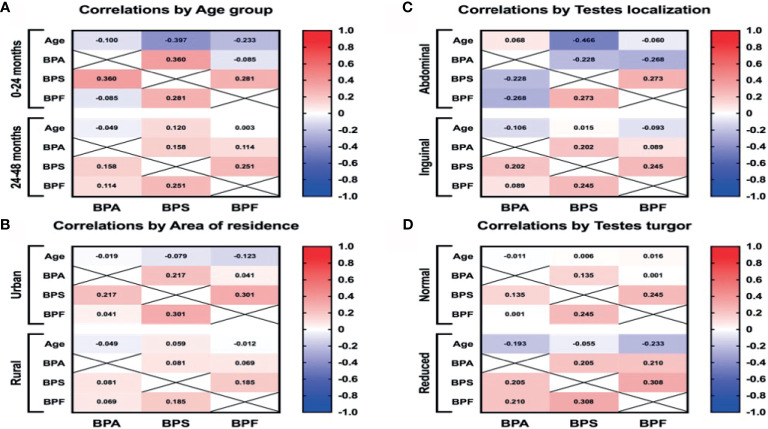
The Pearson correlation between BPs plasma levels and age **(A)**, area of residence **(B)**, intraoperative testes localization **(C)**, and testes turgor **(D)**. Red and blue colors indicate positive and negative correlations, respectively. The deeper the color the more significant is the corresponding correlation.

The purpose was to investigate whether there is a correlation between higher BPs concentration and living in the urban area. In the study group, we found significant differences in BPA levels depending on their place of residence. Boys from urban areas had significantly higher levels of BPA p <0.01 (**Table 2**). Also in children from urban areas, we found a weak positive correlation (r=0.301) between BPS and BPF level (**Figure 3**). The results indicate higher exposure to BPs in urban area.

**Table 2:** Bisphenols plasma levels in the unilateral cryptorchidism and healthy control group in reference to area of living^a^.

Bisphenols (ng/ml)	Urban area	Rural area
**BPA**	16.52 (6.85; 36.99)	7.25 (1.71; 20.15)
**BPS**	4.22 (2.03; 8.35)	3.68 (0.52; 9.54)
**BPF**	3.82 (0.04; 8.07)	3.03 (0.04; 6.18)

a Data presented as median values with 25^th^ and 57^th^ percentile in the brackets.

### Bisphenol Levels Depending on Age

The purpose was to investigate whether there is a correlation between higher BPs concentration and the patient’s older age There were no statistically significant differences between the concentrations of bisphenols and the patients’ age. In the younger group (0-12 months), we found a weak positive correlation (r=0.36) between the levels of BPA and BPS, p<0.01 (**Figure 3**). Generally, the results indicate that the older age of the patient was not associated with a higher exposure to BPs.

### Intraoperative Characteristics

The aim was to examine potential correlation between higher BPs level and testis condition. For testes with reduced turgor, we also noted a weak positive correlation (r=0.308) between the levels of BPS and BPF (**Figure 3**). On the other hand, we did not detect higher plasma concentrations of BPs in boys with reduced testes turgor. Unexpectedly, by evaluating the location of the undescended testes, we observed statistically significant higher levels of BPA in boys with their testes localized in the inguinal region (p<0.01). No correlations were found between plasma BPA, BPS and BPF levels and the size of the undescended testes. The results indicate that higher levels of BPs were not associated with worse testicle condition (smaller size or abdominal localization). On the other hand, higher level of BPS and BPF were connected with reduced testicle turgor.

### Risk Factors of Cryptorchidism

We have not found any correlation between the potential risk factor and incidence of cryptorchidism. All boys were born on time (37-42 weeks) with normal birth weight (range 2500-3500 gram). All mothers denied smoking, drinking alcohol and hormonal treatment during pregnant.

## Discussion

Over the last decades, medical reports have focused on potentially adverse effects of endocrinal chemical compounds on human well-being. Especially the hormonal system, including the reproductive hormones, tends to be notably susceptible to environmental factors. Nowadays, the most widespread EDCs are BPA, phthalates and pesticides (18). What is worse, it appears that exposure to the most common EDCs is inevitable. These chemicals are persistently released into soil and water, then absorbed by microorganisms, plants and animals, and eventually by humans as the final part of the food chain (19). BPA has been studied extensively, and is used universally for the production of polycarbonate plastics, food stuff containers, dental composites and thermal receipts (20, 21).

BPA-free labeled products were expected to be safer, but they often consist of alternative chemicals, e.g. BPS and BPF. To this day, the use of BPS and BPF is unregulated and their tolerable intake values remain unknown (22). BPS was first used in 2005 (23) and it can be found in phenolic resin, cleaning products, or thermal paper, marketed as BPA free (24). Nowadays, BPS is the most common bisphenol analogue (25). It is also used as an electroplating solvent and a component of phenolic resin (23). Moreover, BPS is used in toys and baby bottles, while BPF is detected in products which have to be thick and durable, such as tanks, industrial floors, coatings and electrical varnishes (23). What is worth emphasizing, both analogues are detected in commonly used products: toothpaste, lotions, make-up, body washes, shampoos and conditioners (26), but also, like BPA, in food and daily paper products (e.g. flyers, tickets, airplane boarding passes) (27), even in dust. Similarly to BPA, BPS and BPF have also been detected in water, soil, sediments and sewage effluents (28). Compared to BPA, BPS is more resistant to light and heat, and less biodegradable, and therefore capable of lingering in the environment longer (29).

BPS and BPF have also been found in serum (30), placenta (31), breast milk (32), as well as maternal and cord blood serum (33). According to a recent study (34), EDCs could be harmful also at low-doses and their influence cannot be predicted on the basis of the effects recorded at high doses. Most studies claim that BPS and BPF have similar hormonal activities as BPA (23). A study on zebra fish has shown that BPS is associated with a reduction in gonad weight and alterations in plasma testosterone and estrogen levels (35). In our study, we did not find a correlation between the level of BPS and testicular size. *In vitro* research has found estrogenic activity of BPS, which binds with the human ERα and G-protein coupled receptor 30 (GPR30) (36). BPS also has both androgenic (37) and antiandrogenic activity (38). Similarly, *in vivo* and *in vitro* data shows estrogenic, antiestrogenic, androgenic, antiandrogenic and thyroidogenic activity of BPF (23). The level and duration of exposure to bisphenol A depends on the route of exposure (39). BPA which is swallowed is rapidly metabolized (40). It is eliminated as a conjugated BPA: BPA monosulfate, BPA disulfate and BPA glucoronide (without estrogenic activity) (41). On the other hand, mammalian studies prove that duration of metabolism is significantly extended when phenols are held in the mouth. Absorbed sublingually or from buccal tissues, the substance underwent rapid first-pass metabolism (42). There are many conflicting results regarding the hypothesis that BPA may have a nonlinear, or nonmonotonic, dose–response curve (43). This means that the biological effects of BPA could exist even below exposure levels traditionally defined as no observed adverse effect levels (NOAELs).

Investigations on human exposure to bisphenol analogues are scarce. BPS and BPF have been found in the urine samples of people from the United States and Asian countries (27, 44), and both BPS and BPF have been identified in samples of human urine at concentrations analogous to BPA (27, 44). According to data from the US National Health and Nutrition Examination Surveys (NHANES), in 2013-2016 (1831 children from 8 to 19 years) BPA, BPF and BPS were detected in 97.5%, 55.2% and 87.8% urine samples, respectively (45). Epidemiological data on bisphenol alternatives show that BPA is detected more frequently than BPF and BPS (46). However, in the years following the above study, the authors noticed a downward trend in BPA concentrations and an increase in BPS levels. Nevertheless, the levels of BPA in urine samples are still higher than those of BPS (25). In the present study, in prepubertal boys with unilateral cryptorchidism BPA was detected in 73.2% plasma samples, BPS in 15.1% cases and BPF in 11.7%. Therefore, our results are in line with the mentioned studies.

We noticed that in boys with cryptorchidism from urban areas, the levels of bisphenol were significantly higher than in those inhabiting rural areas. In the former group, we also noticed a weak positive correlation between the levels of BPS and BPF. A study from Turkey (47) has also shown significant differences between males residing in rural and urban areas. Moreover, people living in China, or near e-waste dismantling facilities (48), had significantly higher urinary concentrations of BPA and BPF. Conversely, in a study from South Korea (49), the authors found higher concentrations of urine BPA in residents of rural areas.

### Influence on Cryptorchidism

The etiology of undescended testes is still unclear. Bisphenols have a weak estrogenic activity. Correct natural descent of the male gonads depends on hormones, such as insulin-like peptide 3 (INSL3) and testosterone. Researchers suggest that *—* through non-classical estrogen receptors *—* BPA could affect Leydig cells and hence the secretion of INSL3 (50). It alters the expression of aromatase and 17α‐hydroxylase/17,20 lyase and intrudes into LH receptor‐ligand binding. This negative effect was observed during the “masculinization programming window”, i.e. from 6 to 14 gestational weeks. All bisphenols (A, F,S) decrease the expression of *Lhcgr* (a gene encoding the LH/CG receptor) and almost all the genes which are associated with testosterone synthesis (22). In high concentrations, BPA binds with the androgen receptor (AR) and thus blocks it (29). Similarly, BPF has an influence on human fetal testes, simultaneously reducing testosterone secretion and inducing 17β-estradiol production (22). According to studies from different parts of the world, urinary levels of BPA were higher in pediatric populations than in adults (51–53). Interestingly, there are also some gender differences in urinary BPA levels. Mean levels of bisphenol A in men are higher (52). Anti-androgenic results of BPA could also disturb regulation of testosterone on INSL3 gene expression. An experiment on mice showed that BPA increased germ cell apoptosis (54). On the other hand, Fenichel et al. did not find a significant increase in bisphenol A in cryptorchid children (55, 56). However, mean levels of bisphenol A were higher in nonpalpable compared to palpable gonads. In the present study, higher levels of plasma BPA were observed in patients with the gonads located in the inguinal canal than in those whose gonads were in the abdominal cavity. We did not find significant differences between the location of the gonads and BPS or BPF levels. Similarly, we did not notice significant differences between normal or reduced turgor of undescended testes and concentrations of BPs.

### Oxidative Stress

Oxidative stress could provoke testicular dysfunction in undescended testes. Higher temperature is associated with oxidative stress (57). An increase in the number of reactive oxygen species (ROS) in cryptorchidism was also correlated with a decrease in testosterone level (58). Animal studies have shown that BPA induces oxidative stress in male testes and epididymis (59). Likewise, also bisphenol S and F may induce oxidative stress (60). A study from China also suggests that BPA and BPF exposure is connected with oxidative stress (48). In people living near e-waste dismantling facilities urinary concentrations of oxidative marker: 8-Hydroxy-2′-deoxyguanosine (8-OHdG) were significantly higher and positively correlated with higher urinary concentrations of BPA and BPF.

### Influence on Male Fertility

Although the association between BPA exposure and semen quality, levels of reproductive hormones and fertility of couples, was described in the literature, direct evidence remains limited. These inconsistent results could be explained by several factors, such as target groups, sample sizes, geographical and male seasonal variations in semen parameters. According to some of the experiments, BPA exposure of even less than 50 mg/kg is associated with decreased testosterone levels, both in rats (61, 62) and mice (63). Similarly, lower doses of BPA could cause semen damage, e.g.: sperm DNA damage (64), decreased sperm counts (65) or impaired sperm motility (64, 66). Some authors go even further and call BPA a testicular toxic agent (67). On the other hand, results in male rodents are inconclusive. Even high exposure of adult mice to BPA in utero does not lead to changes in testis weight, sperm production or spermatogenesis (67).

The prevalence of male reproductive disorders has increased in many Western countries (68), particularly, impaired semen quality and testicular cancer. Both disorders, as well as cryptorchidism and hypospadias, are components of the testicular dysgenesis syndrome (TDS), which was first named by Skakkebaek et al. (69). In cross-sectional analysis, urinary bisphenol S was detected in 76% samples and was associated with lower semen parameters (such as lower ejaculate volume, sperm concentration, total count and motility) (70). Another experiment on mice has shown that BPS inhibits testosterone production and, its effect on fetal mouse testes is stronger than this of BPA (36).

Exposure to BPS has been connected with cellular oxidative stress (71) and side effects on the male reproductive system (72). Shi et al. (73) found that mice which had been exposed to BPS from the first gestational period, had worse sperm quality: sperm count was reduced and low sperm motility was noted. Abnormal distribution of spermatogenesis stages in the developing testes was also observed. The first *in vitro* study on human testes (22) reported that BPS, BPF and BPA had decreased spermatogenesis, and BPS was harmful at doses ten times lower than other analogues.

## Conclusion

In this research we provided a characterization of prepubertal boys suffering from unilateral cryptorchidism and exposed to BPA and the two analogues, BPS and BPF. Our study provides novel and unique information about exposure to bisphenols in ediatric populations. We detected all the three bisphenols in our study and control populations, which indicates that exposure to these BPs is extensive and widespread. Living in urban areas could increase the risk of BPA exposure. In summary, exposure to chemicals known as bisphenol A, bisphenol F and bisphenol S is real. Instead of being innovative, safe solutions, the new bisphenols seem to have become yet another health hazard.

## Data Availability Statement

The original contributions presented in the study are included in the article/supplementary material. Further inquiries can be directed to the corresponding authors.

## Ethics Statement

The studies involving human participants were reviewed and approved by Ethics Committee of the Medical University of Bialystok (No R-I-002/288/2017). Written informed consent to participate in this study was provided by the participants’ legal guardian/next of kin.

## Author Contributions

Conception or design of the work: AH. Data collection: EM, MK, and JC. Data analysis and interpretation: JH and KG. Drafting the article: MK. Critical revision of the article: EM. Final approval of the version to be published: AH and WD. All authors provide approval for publication of the content, agree to be accountable for all aspects of the work in ensuring that questions related to the accuracy or integrity of any part of the work are appropriately investigated and resolved. All authors contributed to the article and approved the submitted version.

## Conflict of Interest

The authors declare that the research was conducted in the absence of any commercial or financial relationships that could be construed as a potential conflict of interest.
